# Diagnostic and prognostic implications of ribosomal protein transcript expression patterns in human cancers

**DOI:** 10.1186/s12885-018-4178-z

**Published:** 2018-03-12

**Authors:** James M. Dolezal, Arie P. Dash, Edward V. Prochownik

**Affiliations:** 1 Division of Hematology/Oncology, Children’s Hospital of Pittsburgh of UPMC, Pittsburgh, PA USA; 2 Department of Microbiology and Molecular Genetics, The University of Pittsburgh Medical Center; The University of Pittsburgh Hillman Cancer Center, Pittsburgh, PA USA

**Keywords:** Diamond-Blackfan anemia, Shwachman-diamond syndrome, *t*-SNE, Ribosomopathy, 5q- syndrome

## Abstract

**Background:**

Ribosomes, the organelles responsible for the translation of mRNA, are comprised of four rRNAs and ~ 80 ribosomal proteins (RPs). Although canonically assumed to be maintained in equivalent proportions, some RPs have been shown to possess differential expression across tissue types. Dysregulation of RP expression occurs in a variety of human diseases, notably in many cancers, and altered expression of some RPs correlates with different tumor phenotypes and patient survival. Little work has been done, however, to characterize overall patterns of RP transcript (RPT) expression in human cancers.

**Methods:**

To investigate the impact of global RPT expression patterns on tumor phenotypes, we analyzed RPT expression of ~ 10,000 human tumors and over 700 normal tissues from The Cancer Genome Atlas (TCGA) using *t*-distributed stochastic neighbor embedding (*t*-SNE). Clusters of tumors identified by *t*-SNE were then analyzed with chi-squared and *t*-tests to compare phenotypic data, ANOVA to compare individual RPT expression, and Kaplan-Meier curves to assess survival differences.

**Results:**

Normal tissues and cancers possess distinct and readily discernible RPT expression patterns that are independent of their absolute levels of expression. In tumors, RPT patterning is distinct from that of normal tissues, identifies heretofore unrecognized tumor subtypes, and in many cases correlates with molecular, pathological, and clinical features, including survival.

**Conclusions:**

RPT expression patterns are both tissue-specific and tumor-specific. These could be used as a powerful and novel method of tumor classification, offering a potential clinical tool for prognosis and therapeutic stratification.

**Electronic supplementary material:**

The online version of this article (10.1186/s12885-018-4178-z) contains supplementary material, which is available to authorized users.

## Background

Eukaryotic ribosomes are among the most highly evolutionarily conserved organelles, comprised of four ribosomal RNAs (rRNAs) and approximately 80 ribosomal proteins (RPs). Responsible for translating mRNA into proteins, ribosomes were long believed to be nonspecific “molecular machines” with unvarying structures and function in different biological contexts. Recent evidence has shown, however, that some individual RPs are expressed in tissue-specific patterns and can differentially contribute to ribosome composition, affect rRNA processing, and regulate translation [1]. Despite the complexity of RP assembly in ribosomes, early studies of ribosome function revealed that the catalytic activity responsible for peptide bond formation might depend only on the presence of rRNAs and a small number of core RPs [2]. This finding, in conjunction with the observation that some RPs are expressed in a tissue-specific manner, has led to speculation that one purpose for the evolutionary emergence of RPs may have been to confer translational specificity and adaptability [1, 3].

An increasing body of evidence continues to show that RPs do, in fact, have important roles in imbuing ribosomes with mRNA translational specificity. During embryonic development, RPs are expressed at different levels across tissue types, and loss of RPs due to mutation or targeted knockdown produces specific developmental abnormalities in plants, invertebrates, and vertebrates. The tissue-specific patterning that occurs as a consequence of individual RP loss suggests that some RPs serve to guide the translation of specific subsets of transcripts in order to influence cellular development. While the mechanism(s) by which RPs confer translation specificity are not entirely known, one may involve the alteration of ribosome affinity for transcripts with specific *cis*-regulatory elements, including internal ribosome entry sites (IRES) elements and upstream open reading frames (uORFs) [1].

RPs also participate in a variety of extra-ribosomal functions. In normal contexts, ribosome assembly from individual rRNAs and RPs is a tightly regulated process, with unassembled RPs undergoing rapid degradation. Disruption of ribosomal biogenesis by any number of extracellular or intracellular stimuli induces ribosomal stress, leading to an accumulation of unincorporated RPs. In some cases, these free RPs may participate in a variety of extra-ribosomal functions, including the regulation of cell cycle progression, immune signaling, and cellular development. Many free RPs bind to and inhibit MDM2, a potentially oncogenic E3 ubiquitin ligase that interacts with and promotes the degradation of the TP53 tumor suppressor. The resulting stabilization of TP53 triggers cellular senescence or apoptosis in response to the inciting ribosomal stress. Additional extra-ribosomal functions of RPs are numerous, and have been recently reviewed [4, 5].

Given their role in regulating gene translation, cellular differentiation, and organismal development, it is perhaps unsurprising that altered RP expression has been implicated in human pathology. Indeed, an entire class of diseases has been shown to be associated with haploinsufficient expression or mutation in individual RPs. These so-called “ribosomopathies,” including Diamond-Blackfan Anemia (DBA) and Shwachman-Diamond Syndrome (SDS), are characterized by early onset bone marrow failure, variable developmental abnormalities and a life-long cancer predisposition that commonly involves non-hematopoietic tissues [6, 7]. The loss of proper RP stoichiometry and ensuing ribosomal stress result in increased ribosome-free RPs, which bind to MDM2 and impair its ubiquitin-mediated degradation of TP53 [6, 8–10]. The resulting TP53 stability is believed to underlie the bone marrow failure that affects the erythroid or myeloid lineages in DBA and SDS, respectively. The developmental abnormalities of the ribosomopathies are variable and associate with specific RP loss or mutation. For example, RPL5 loss in DBA is specifically associated with cleft palate and other craniofacial abnormalities whereas RPL11 loss is associated with isolated thumb malformations [11].

Ribosomopathy-like properties have also been observed in various cancers. We have recently shown that RP transcripts (RPTs) were dysregulated in two murine models of hepatoblastoma and hepatocellular carcinoma (HCC) in a tumor-specific manner and in patterns unrelated to tumor growth rates [12]. These murine tumors also displayed abnormal rRNA processing and increased binding of free RPs to MDM2, reminiscent of the aforementioned inherited ribosomopathies.

Perturbations of several individual RPs have been found in numerous human cancers, including those of the breast, pancreas, bladder, brain and many other tissues [13–25]. Mutations and deletions of RP-encoding genes have also been found in endometrial cancer, colorectal cancer, glioma, and various hematopoietic malignancies [26–28]. Indeed, the Chr. 5q- abnormality associated with myelodysplastic syndrome and the accompanying haploinsufficiency of RPS14 is considered one of the prototype “acquired” ribosomopathies that are often classified together with DBA, SDS and other inherited ribosomopathies [6]. Although many free RPs can induce cellular senescence during ribosomal stress via the MDM2-TP53 pathway, not all RPs possess such tumor suppressor functions. RPS3A overexpression, for example, actually transforms NIH3T3 mouse fibroblasts and induces tumor formation in nude mice [29].

A recent attempt to summarize the heterogeneity of RPT expression in human cancers was limited to describing expression differences of single RPTs among cancer cohorts, without accounting for larger patterns of variation that might better distinguish tumors from one another [3]. RPT expression patterns were, however, examined in normal tissues using the dimensionality-reduction technique Principal Component Analysis (PCA) in the aforementioned study. These results provided hints of cell-specific patterning in the hematopoietic tissues examined, but not all cell types clustered into obviously distinct groups.

In the current work, we leverage a machine learning technique known as *t-*distributed stochastic neighbor embedding (*t*-SNE) to identify distinct patterns of global RPT expression across both normal human tissues and cancers. Like PCA, *t-*SNE is a dimensionality reduction technique used to visualize patterns in a data set [30]. With either technique, patterns shared between data points are represented with clustering. However, *t*-SNE differs from PCA in that it performs particularly well with highly dimensional data and is able to distinguish non-linear relationships and patterns. With this technique, we show that virtually all normal tissues and tumors can be reliably distinguished from one another based on their signature RPT expression patterns. Tumors differ from normal tissues, but retain sufficient remnants of normal tissue patterning to allow for their origin to be easily discerned. Finally, we show that a number of cancers possess subtypes of RPT expression patterns that correlate in readily understandable ways with molecular markers, various tumor phenotypes, and survival.

## Methods

### Accessing ribosomal protein transcript expression data

RNA-seq whole-transcriptome expression data for 9844 tumors and 716 normal tissues from The Cancer Genome Atlas (TCGA) was accessed using the University of California Santa Cruz (UCSC) Xenabrowser [31]. Only primary tumors were included for analysis, apart from the melanoma (SKCM) cohort, as the vast majority of tumors with sequencing data in this cohort were metastatic (78%). The total number of samples analyzed in each cohort can be found in Additional file 1: Table S1. For each of the 30 cancer cohorts, RNA-seq data was selected according to the label “gene expression RNAseq (polyA+ IlluminaHiSeq).” “IlluminaGA” RNA-seq expression data was used for the cohort Uterine Corpus Endometrial Carcinoma (UCEC), as this group of data had more samples than the “IlluminaHiSeq” group. For all cancer cohorts, expression data for 80 cytoplasmic RP genes were extracted and base-two exponentiated, as the raw RPKM (Reads Per Kilobase per Million mapped reads) expression data was stored log-transformed. The sum of total RPKM counts for all RP genes was calculated for each sample, and relative expression of each RP gene in a sample was calculated by dividing the RPKM gene expression by this summed expression.

### Visualizing ribosomal protein transcript expression

Principal component analyses and *t*-SNE analyses of RPT relative expression in normal tissues and tumor samples were performed using TensorFlow r1.0 and Tensorboard [32]. *t*-SNE analyses were performed at a learning rate (epsilon) of 10 with 5000 iterations or until the visualization stabilized. *t*-SNE was initially performed in two dimensions for all analyses; data sets that could not be cleanly visualized with two dimensions, particularly those with a large number of samples, were visualized with three-dimensional *t*-SNE. Multiple analyses were performed with perplexity settings varying between 6 and 15 for all individual cohort analyses and 10–30 for all grouped cohort analyses, with final perplexity settings for each analysis chosen to maximize cluster distinctions. Clusters of at least 10 samples which distinctly separated visually from other samples were named and samples from these clusters were identified. 3D area maps of RPT relative expression were generated using Microsoft Excel, with each sample listed across the x-axis, RPTs listed across the z-axis, and relative expression of each RPT across the y-axis.

### Comparing *t*-SNE clusters

Relative expression of RPTs were compared between *t*-SNE clusters with Analyses of Variance (ANOVA) using R version 3.3.2 [33]. ANOVA *p*-values were log_10_-transformed and used to generate Volcano plots comparing expression patterns between clusters. Volcano plots were graphed with Graphpad Prism 7 (GraphPad Software, Inc., La Jolla, CA).

Clinical and survival data for each TCGA cancer cohort were accessed again using the UCSC Xenabrowser under the data heading “Phenotypes.” For each cohort, survival curves of tumors in each *t*-SNE cluster were compared with Mantel-Haenszel (log-rank) and Gehan-Breslow-Wilcoxon methods using Graphpad Prism 7. Categorical clinical variables were compared between clusters of tumors with chi-squared tests. Continuous variables which were normally distributed were compared with *t*-tests assuming heteroskedasticity, and non-normally-distributed variables were compared with Wilcoxon sign-rank tests. All statistical tests were two-tailed.

### Co-regulated RPTs

Certain groups of RPTs possessed recurring, highly-significant differences between multiple *t*-SNE clusters, including *RPL3*, *RPL8*, *RPS4X*, and *RPL13*. For each TCGA cohort with a cluster that possessed significantly different relative expression of one of these transcripts, relative expression of all other RPTs was compared between the identified cluster and other tumors in the same cohort. Co-regulated transcripts were defined as those with consistent differences in relative expression when comparing clusters of interest to other tumors from the same cohort (Table 1). For example, five TCGA cohorts had a *t*-SNE cluster with significant relative overexpression of *RPL8* and *RPL30*. When comparing relative expression of other RPTs between these clusters and other tumors from the same cohorts, all five clusters with high *RPL8* and *RPL30* also displayed, on average, lower relative expression of *RPL10* and higher relative expression of *RPL7.*

**Table 1:** Recurring patterns of RPT expression in tumors across cancer cohorts

Primary Difference	Co-regulated RPTs		Observations	
	*Lower expression*	*Higher Expression*	*Cohort*	*Cluster*
Low *RPL3*	*RPL5, RPL10, RPL17, RPL22, RPL26, RPS23*	*RPL28, RPL35, RPL36, RPL38, RPLP2*	THCA^a^	1
			GBMLGG	3, 4
			LIHC	2, 3
			KIRC	3
			THYM	2
			PRAD	2, 3
			PAAD	1
			PCPG	1
			DLBC	2
High *RPL8* and *RPL30*	*RPL10, RPL13A, RPL19, RPL21, RPL34, RPL37A, RPL4, UBA52, RPL5, RPS11, RPS12, RPS13, RPS17, RPS27A, RPS8, RPS9*	*RPL36A, RPL7, RPS20*	BRCA	3
			LIHC	3
			PRAD	2
			LUNG	1
			SKCM	2
			HNSC	2
High *RPS4X*	*RPL13, RPL23, RPL37, RPL8, RPLP1, RPS16, RPS2*	*RPS3A, RPL17*	PRAD	1
			KIRC	1
			THCA	1, 2
			STAD	2
			LAML	1
			CESC	2
High *RPL13*	*RPL10, RPL10A, RPL26, RPL3, RPL35A, RPL41, RPL5, RPL7, RPL7A, RPS12, RPS13, RPS14, RPS15A, RPS18, RPS23, RPS27, RPS3, RPS3A, RPS4X, RPS5*	*RPL12, RPL13, RPL18, RPL24, RPL27A, RPL28, RPL32, RPL35, RPL36, RPL37, RPL38, RPL39, RPLP1, RPLP2, RPS15, RPS17, RPS19, RPS20, RPS24, FAU, RPS4Y1, RPS9*	PRAD	3
			UCEC	3
			KIRC	3

Four recurring patterns of expression distinguishing tumor clusters from one another were observed in multiple clusters across cancer cohorts, as shown in Figs. 2 and 3a. For each pattern, the most significant and defining RPT expression difference is listed under “primary difference.” Other significant RPT expression differences associated with these patterns are listed under “co-regulated RPTs.” “Low” and “high” expression is defined relative to other tumors within a cancer cohort. Clusters with the described pattern are listed under “observations”

^a^While tumors in this cluster had relatively lower expression of *RPL3*, other RPTs were not co-regulated in the same manner

### RP gene copy number variations (CNVs)

CNV data for TCGA tumors was accessed using the UCSC Xenabrowser under the data heading “copy number (gistic2_thresholded).” Positive values were classified as amplifications, and negative values were classified as deletions. The frequency of amplifications and deletions in RP genes were compared between clusters of tumors in each TCGA cohort using chi-squared tests and adjusted for 5% false discovery rate. Within each cancer cohort, clusters of tumors with significantly greater incidence of a CNV compared to other tumor clusters, and which possessed > 90% incidence of this copy number variation, were included in Table 2.

**Table 2:** RP gene copy number alterations associated with *t*-SNE clusters

Genes	Type	Location	Cohort	Cluster	Frequency
*RPL19, RPL23*	Amplification	17q12	BRCA	1	98.9%, 83.1%
*RPL8, RPL30*	Amplification	8q22	BRCA	3	100%, 97.5%
			LIHC	3	98.8%, 98.8%
			PRAD	2	95.1%, 97.5%
			LUNG	1	100%, 94.3%
			SKCM	2	94.2%, 88.5%
			HNSC	2	93.2%, 85.3%
*RPS3*	Amplification	11q13	BRCA	2	100%
			LUNG	5	90.5%
*RPS16*	Amplification	19q13	LUNG	6	100%
*RPL13A, RPL18, RPL28, RPS5, RPS9, RPS11, RPS16, RPS19*	Deletion	19q13	GBMLGG	5	98.8% - 99.4%
*RPL11, RPL22, RPL5, RPS8*	Deletion	1p	GBMLGG	5	97.5%
*RPS24*	Deletion	10q22	GBMLGG	3	90.5%

Some tumor clusters were significantly associated with greater incidence of copy number alterations than other tumors from the same cancer cohorts (α <  0.01); clusters with > 90% of tumors possessing a given copy number alteration are included in this table. Cancer cohorts with no tumor clusters strongly associating with a RP copy number alteration were excluded from this table

### Classification models

Using RPT relative expression in tumors and normal tissues, classification models were created using both logistic regression and feed-forward, fully-connected artificial neural networks (ANNs) [34]. LR models were used for binary classifiers and developed with Stata SE 14 (StataCorp LP, College Station, TX) with *c*-statistics, sensitivity, and specificity reported in Additional file 1: Table S2. ANN models were generated for classifiers with multiple outcomes (e.g. tissue of origin models) and binary classifiers with a LR model that failed to converge.

ANN models were created and tested using TensorFlow with graphics processing unit (GPU) acceleration on a Titan X Pascal (NVIDIA, Inc. Santa Clara, CA). To reduce bias, samples were balanced for both training and testing by cancer cohort such that each training and test set had the same number of samples from each cohort. 60% of data sets were used for training and 10% for validation and hyper-parameter tuning. Hyper-parameter sweeps were used to test all possible combinations of the following: learning rate (0.001, 0.002, 0.005, 0.01), batch size (100, 500, none), dropout rate (0.9, 0.95, 1), hidden layer structure (both one and two layers with sizes varying between 0 and 200 in increments of 25), and L2 regularization rate (0.00001, 0.0001, 0.001). All ANNs utilized ReLU activation functions. Neural network training performance was monitored with Tensorboard and stopped once validation accuracy had plateaued. The remaining 30% of data comprised a separate test set, which was used to test the final model’s classification accuracy once the hyper-parameters were chosen and the model trained. Performance of ANN models on the separate test sets were reported as classification accuracies in Additional file 1: Table S2.

## Results

### t-SNE identifies tissue- and tumor-specific RPT expression

**Fig. 1:**
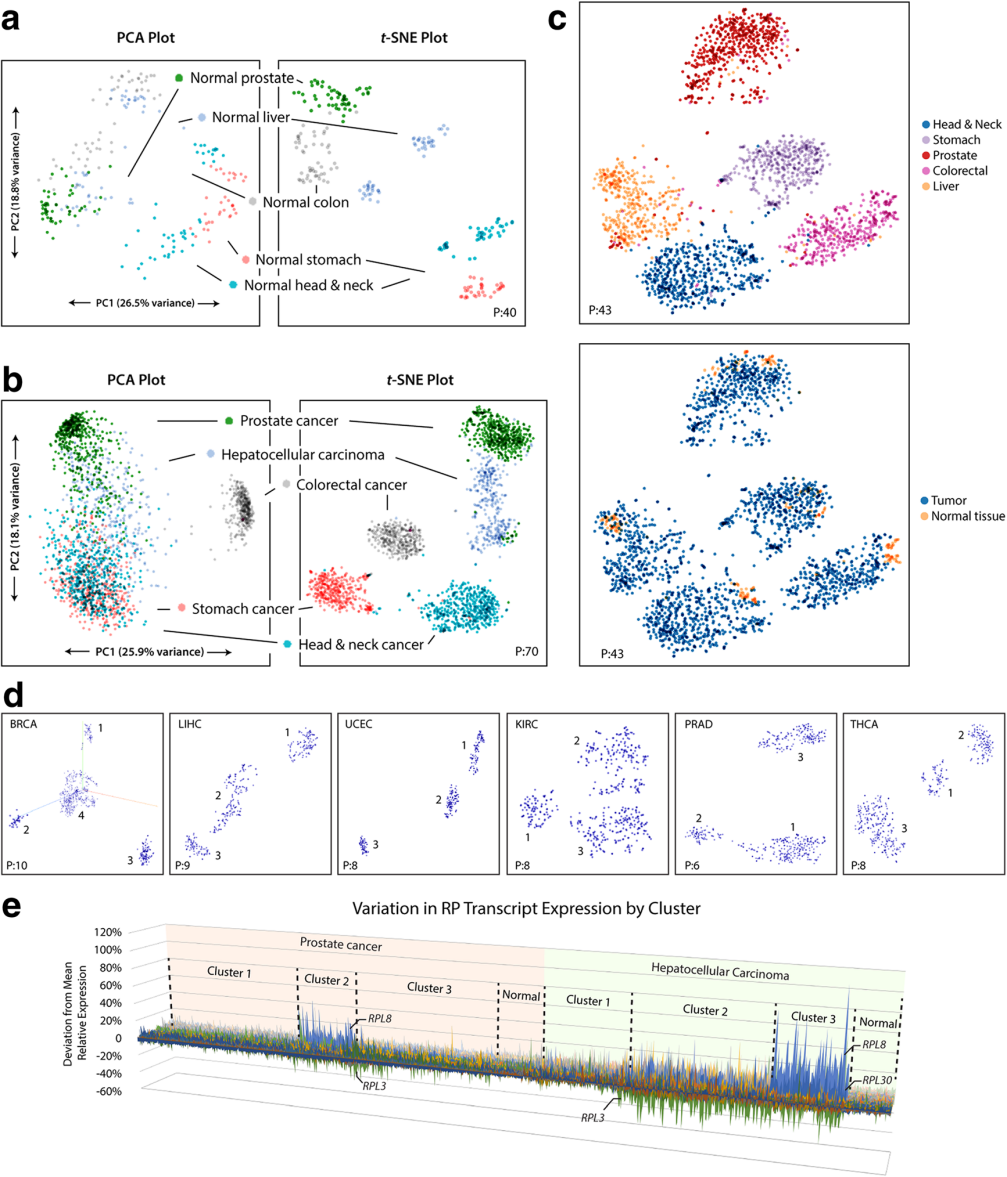
*t*-SNE better identifies clusters of RPT expression than PCA. **a**. Relative expression of RPTs in normal tissues from five cohorts was analyzed with PCA. In both methods, clustering occurs when samples possess similar underlying patterns of variation. *t*-SNE provides more distinct clusters that better associate with tissue of origin, indicating that normal tissues have distinct patterns of RPT expression. Axes are not labeled with *t*-SNE, as points are not mapped linearly and axes are not directly interpretable. **b**. Similar analyses in tumors. PCA clusters are poorly defined and do not correlate strongly with tumor type. *t*-SNE clusters are distinct and strongly associate with cancer type, indicating that tumors possess unique patterns of RPT expression based on their tissue of origin. **c**. Combined *t*-SNE analysis of RPT expression in normal tissue and tumor samples. Normal tissues and tumors cluster together but can be distinguished from one another, indicating that the latter retain a pattern of RPT expression resembling that of the normal tissue from which they originated. **d.** Many single cancer cohorts demonstrate sub-clustering by *t*-SNE. Clustering of six cohorts are provided as examples here. The number of clusters found in each cohort is listed in Additional file 1: Table S1. **e**. 3D area map of RPT relative expression in tumors from two cancer cohorts, sorted by cluster. The x-axis represents individual tumors, the z-axis represents individual RPTs, and the y-axis represents deviation from the mean relative expression. Cluster 2 of prostate cancer and Cluster 3 of HCC are both comprised of tumors with high relative expression of *RPL8* and low *RPL3*. See Additional file 1: Figure S1, S2, and S5 for additional *t*-SNE plots of tumors and normal tissues. Perplexity settings for *t*-SNE analyses are designated in each plot by “P:”. For all analyses, learning rate (epsilon) = 10 and iterations = 5000

RNA-seq expression data for 9844 tumors (30 cancer types) and 716 matched normal tissues were obtained from The Cancer Genome Atlas (TCGA) [35]. Relative expression of RPTs was calculated for all samples and first analyzed using PCA. To a modest degree, normal tissue samples could be distinguished by their RPT expression patterns, though many tissue types demonstrated considerable overlap (Fig. 1a and Additional file 1: Figure S1A). Patterns of RPT expression in tumors were even more heterogeneous, and most cancer cohorts did not cluster discretely (Fig. 1b).

Samples were then analyzed with *t*-SNE, which more clearly identified clusters of variation due to its ability to identify non-linear relationships among RPTs (Fig. 1a and b and Additional file 1: Figure S1B). Clustering of normal tissue samples correlated nearly perfectly with tissue type. Tumors also demonstrated clustering that strongly associated with tissue type, with 20 cohorts segregating into largely distinct and non-overlapping groups. When both normal tissues and tumors were analyzed together with *t*-SNE, samples also generally grouped into large clusters according to tissue type. Normal tissues, however, localized into smaller sub-clusters distinct from tumors (Fig. 1c and Additional file 1: Figure S2). Thus, while samples nearly always possessed RPT expression patterns specific to their tissue type, normal tissues and tumors could be readily distinguished from one another.

Five cancer cohorts, including cholangiocarcinoma (CHOL), lung (LUNG), bladder (BLCA), cervical (CESC), and uterine carcinosarcoma (UCS), were comprised of tumors that lacked tissue-specific RPT expression profiles and did not group into distinct clusters. These tumors displayed significant overlap with each other as well as with tumors from the remaining five cohorts – liver HCC (LIHC), colorectal (COADREAD), mesothelioma (MESO), pancreatic (PAAD), and skin cutaneous melanoma (SKCM) – which otherwise clustered distinctly from one another (Additional file 1: Figure S3). Additionally, two clusters of tumors were found that did not associate with tissue of origin (Additional file 1: Figure S4). The first contained 143 tumors from 15 cohorts, 98% of which had amplification and relative up-regulation of *RPL19*, *RPL23*, and *ERBB2* (Her2/Neu). The second contained 77 tumors from 12 cohorts with no clearly discernable or unifying RPT expression pattern.

### *t*-SNE identifies sub-types of RPT expression within cancer types

Analyzed individually, 19 of 30 cancer types demonstrated sub-clustering of RPT expression with *t*-SNE (Fig. 1d, Additional file 1: Figure S5, and Additional file 1: Table S1). Graphing RPT relative expression by cluster using a 3D area map illustrated the different patterns of expression detected by *t*-SNE (Fig. 1e). In some cases, these clusters differed from one another in the expression pattern of numerous RPTs, as seen with Clusters 1 and 3 of prostate cancer. In other cases, expression patterns appeared to be dominated by the differential relative expression of one or two RPTs, as seen with prostate cancer Cluster 2 and HCC Cluster 3, both of which possess tumors that overexpress *RPL8* and under-express *RPL3* (Fig. 1e). While all clusters were distinct from normal tissues (Fig. 1c and Additional file 1: Figure S2), some were more similar to normal tissues than others, such as prostate cancer Cluster 1 and HCC Cluster 1 (Fig. 1e).

### Classification models

While *t*-SNE analyses are useful for visualization and pattern discovery, they do not alone provide a direct means for classification of future samples. Thus, with the knowledge that RPTs have both tissue- and tumor-specific expression patterns, we constructed various tumor classifier models based on these patterns. The constructed models consisted of both artificial neural network (ANN) and logistic regression [35] classifiers, and are listed in Additional file 1: Table S2. An ANN model classified tumors by RPT content according to their tissue of origin on a separate test set with 93% accuracy. Similarly, a LR model distinguished tumors from normal tissues with > 98% accuracy. Other LR models could distinguish glioblastoma multiforme tumors from other brain cancers with 100% accuracy and were able to stratify both uterine and kidney clear cell tumors according to prognostic group with > 95% accuracy.

### Characterizing tumor clusters identified by t-SNE

**Fig. 2:**
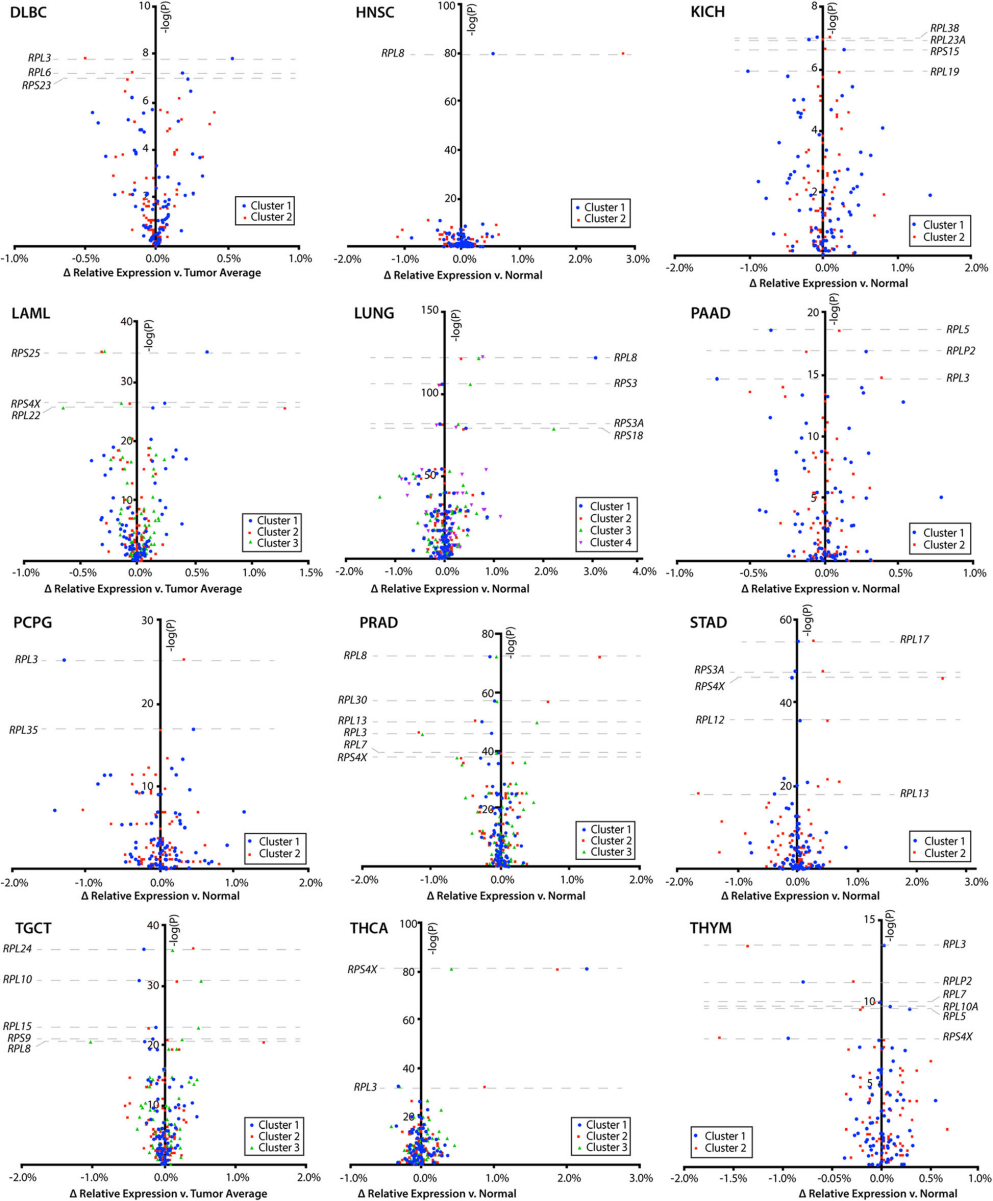
Volcano plots of relative RPT expression in tumor clusters in twelve cancer cohorts. Relative expression of RPTs was compared between tumor clusters in each included cancer cohort with ANOVA tests. The negative log of the ANOVA *P*-value for each RPT is displayed on the y-axis and the difference in relative expression across tumor clusters is displayed on the x-axis. RPTs near the top of the graphs are most significantly differentially expressed between tumor clusters. Note that nearly every RPT in virtually all cancer cohorts falls above –log(P) of 2, corresponding to *P* <  0.01 and indicating that tumor clusters have significantly distinct expression of virtually all RPTs. For each cohort, the number of samples in each cluster are shown under the label “*n*”. Additional volcano plots of seven other cancer cohorts are continued in Fig. 3

**Fig. 3:**
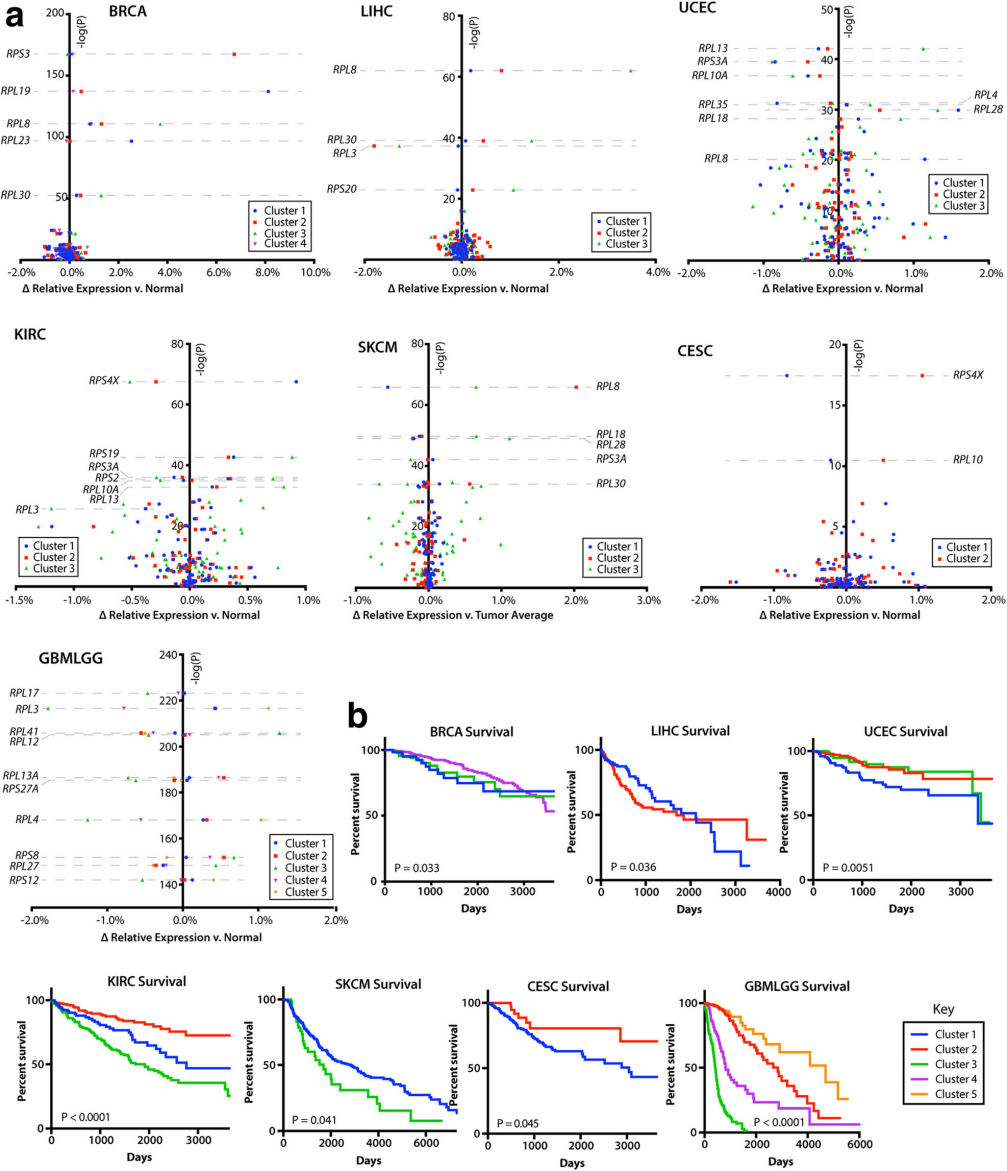
Volcano plots of relative RPT expression in tumor clusters associated with survival. **a.** Volcano plots comparing RPT relative expression between tumor clusters were generated, as in Fig. 2, for the remaining seven cancer cohorts which possessed tumor sub-clustering by *t*-SNE. Note that for the sake of clarity, clusters 5 and 6 are excluded from the LUNG cohort plot. These clusters correlated near perfectly with amplification and highly significant up-regulation of *RPS3* and *RPS16*, respectively (Table 2). **b.** Patient survival by *t*-SNE cluster. Of the 19 cancer cohorts with sub-clustering of RPT expression patterns by *t*-SNE, seven possessed clusters that correlated with survival. Significance was determined with log-rank and Wilcoxon rank sum tests where appropriate, using all survival data available, including any data points beyond what are displayed in the survival curves

In order to quantify the differences in RPT expression that exist between clusters of tumors identified by *t*-SNE, RPT relative expression was compared among clusters of tumors with Analysis of Variance (ANOVA) and graphed with volcano plots (Figs. 2 and 3a). Small but highly significant differences in

the expression of dozens of RPTs occurred in nearly every tumor cluster (P as low as 10^− 220^). As was the case with prostate cancer and HCC, these expression patterns were often dominated by particularly significant differences in expression of one or two RPTs, most commonly *RPL3*, *RPS4X*, *RPL8*, *RPL30*, and *RPL13*. Other tumor clusters, notably those involving the uterus, brain, and lung, possessed more complex differences involving larger numbers of RPTs (Figs. 2 and 3a).

Several recurrent alterations in RPT expression were found among the 19 cancer cohorts with sub-clustering (Table 1). Nine of these cancer clusters, arising from thyroid, brain, liver, kidney clear cell, thymoma, prostate, pancreatic, pheochromocytoma and paraganglioma, and B-cell lymphoma, contained tumors with low relative expression of *RPL3*. These clusters also shared expression patterns with other RPTs, including the relative down-regulation of *RPL5* and up-regulation of *RPL36* and *RPL38*. Excluding thyroid cancers, all other tumor clusters with low *RPL3* also shared 11 other similarly co-regulated RPTs. Additionally, six cancer cohorts – prostate, breast, liver, lung, melanoma, and head and neck – contained tumor clusters distinguished by overexpression of *RPL8*, *RPL30* and *RPS20*, with shared expression patterns of 19 other RPTs. Relative up-regulation of *RPS4X* occurred in tumors from six cohorts, all of which showed similar co-expression patterns of nine other RPTs. Finally, tumor clusters overexpressing *RPL13* were found in prostate, uterine and kidney clear cell carcinoma and shared similar patterns of expression of 42 other RPTs (Figs. 2 and 3a and Table 1).

In some cases, RP gene copy number variations (CNVs) were associated with and could explain the observed clustering (Table 2). Notably, the aforementioned *RPL8*/*RPL30* overexpression pattern strongly correlated with co-amplification of a region on 8q22–24 containing genes encoding these two RPs as well as the Myc oncoprotein and PVT1, a long-non-coding RNA (lncRNA) with oncogenic properties [36, 37]. Similarly, an amplicon containing *RPL19*, *RPL23*, and *ERBB2* (Her2/Neu) was amplified in 99% of the breast cancers in Cluster 1. Some tumor clusters associated with specific CNVs to a lesser degree. For example, 48% of tumors in kidney clear cell carcinoma Cluster 3 possessed deletions of *RPL12*, *RPL35*, and *RPL7A* on 9q33–34. Similarly, half of brain cancers in Cluster 1 possessed a 1p/19q13 co-deletion, compared to nearly 100% of tumors in Cluster 5 with this deletion (Table 2). Other tumor clusters in various cancer cohorts had differences in overall CNV frequencies. In testicular cancer, 39 RP genes were amplified at different frequencies among the three clusters. Endometrial cancer Cluster 1 and HCC Cluster 2 had more CNVs overall, but no RP gene was amplified or deleted with a frequency of greater than 65% in any given tumor cluster.

Many tumor clusters – each representing a distinct RPT expression pattern - significantly associated with various clinical parameters, molecular markers, and tumor phenotypes (Table 3). This was particularly true for brain, testicular, thyroid, lung, and endometrial cancers. Tumor clusters in HCC and head and neck cancers strongly correlated with etiologically-linked infections. For example, chronic hepatitis B infection was two-fold more common in HCC patients with Cluster 2 tumors compared to other HCC patients. Similarly, chronic HPV infection was 4.7-fold more frequent in head and neck cancer patients with Cluster 1 tumors compared to other patients in this cohort. Patient gender also associated with tumor clustering to varying but significant degrees in kidney clear cell carcinoma and AML. Notably, these clusters also associated with differential relative expression of the X-chromosome encoded *RPS4X*. Other clinical markers and tumor phenotypes significantly associated with tumor clustering can be found in Table 3.

**Table 3:** Tumor phenotypes and clinical parameters associated with *t*-SNE clustering

Feature	Cluster	Comparison	P-value
*Breast cancer*			
Her2/Neu status	Cluster 1–88.2%	Other tumors – 7.7%	< 0.0001
*Hepatocellular carcinoma*			
Hepatitis B	Cluster 2–38.4%	Other tumors – 18.6%	< 0.0001
*Endometrial carcinoma*			
Type II (serous) type	Cluster 1–32.4%	Other tumors – 5.2%	< 0.0001
Locoregional disease or recurrence	Cluster 1–8.6%	Other tumors – 2.2%	0.004
*Lung cancer*			
Adenocarcinoma	Cluster 2–80.0%	Other tumors – 37.8%	< 0.0001
Squamous cell carcinoma	Cluster 4–73.6%	Other tumors – 34.2%	< 0.0001
*Kidney clear cell carcinoma*			
Gender	Cluster 1–92.9% female	Other tumors – 29.0% female	
Longest dimension	Cluster 2–1.5 cm	Other tumors – 1.8 cm	< 0.0001
Histologic grade 3 or 4	Cluster 3–69.0%	Other tumors – 41.5%	< 0.0001
Pathologic stage III or IV	Cluster 3–58.5%	Other tumors – 25.5%	< 0.0001
*Thyroid carcinoma*			
Gender	Cluster 3–35.4% male	Other tumors – 11.3% male	< 0.0001
Tall cell subtype	Cluster 1–17.2%	Other tumors – 5.03%	0.0001
Follicular subtype	Cluster 3–27.7%	Other tumors – 10.5%	< 0.0001
*Acute myeloid leukemia*			
Gender	Cluster 1–100% female Cluster 3–81.5% male	Cluster 2–53.6% female	< 0.0001
Complex (> 3 distinct) cytogenetic abnormalities	Cluster 3–21.0%	Other tumors – 5.5%	0.0025
*Testicular cancer*			
Seminoma subtype	Cluster 1–100%	Other tumors – 14.5%	< 0.0001
Embryonal carcinoma subtype	Cluster 2–94.6%	Other tumors – 20.6%	< 0.0001
Teratoma subtype	Cluster 3–84.0%	Other tumors – 14.7%	< 0.0001
*Head and neck cancer*			
HPV infection	Cluster 1–46.1%	Other tumors – 9.8%	0.0001
*Skin cutaneous melanoma*			
Breslow depth	Cluster 1–4.8 cm	Other tumors – 7.6 cm	0.0083
*Brain cancer*			
Glioblastoma	Cluster 3–100%	Other tumors – 0%	< 0.0001
Astrocytoma low-grade glioma	Cluster 2–53.0% Cluster 4–62.7%	Other tumors – 2.5%	< 0.0001
Non-astrocytoma low-grade glioma	Cluster 1–86.7% Cluster 5–97.0%	Other tumors – 29.1%	< 0.0001

Tumor phenotypes and clinical markers were compared between tumor clusters using chi-squared tests, with significance defined as α < 0.01. “Other tumors” are comprised of all tumors from the same cancer cohort not falling into the given cluster. Data were obtained using the UCSC Xenabrowser, under the data heading “Phenotypes.” Cancer cohorts with no tumor clusters significantly associating with any clinical parameter were excluded from this table

Tumor clusters were often predictive of survival, including some clusters that did not significantly associate with any other known tumor subtype (Fig. 3b). For example, Clusters 2 and 4 of the brain cancer cohort, which could not otherwise be distinguished by any known clinical parameter or tumor subtype, possessed vastly different survival patterns. Other cancer cohorts with significant survival differences among clusters included breast, liver, endometrial, kidney clear cell, melanoma, and cervical cancers.

## Discussion

By investigating expression patterns of individual RPTs and utilizing more traditional and less powerful linear forms of dimensionality reduction such as PCA, previous studies have found modest evidence of tissue-specific patterning of RPT expression in some normal tissues and even less evidence in malignant tumors [3]. The failure to reproducibly identify recurrent and convincing patterning is presumably due to the complex regulation of RPT expression and the fact that many of the RPT relationships are non-linear. As shown here, however, the machine learning algorithm *t*-SNE provides a more elegant and robust dimensionality reduction that better highlights the distinct underlying patterns of RPT expression in both tumors and the normal tissues from which they originate.

Consistent with the more restricted and tentative conclusions of previous findings, our results using *t*-SNE clearly demonstrate that RPT expression patterns are not only tissue-specific but provide the ability to define tissue and tumor differences with a heretofore unachievable degree of resolution. The small cluster of 77 neoplasms that did not associate with their respective tissue clusters (Additional file 1: Figure S4) may represent either a subset of tumors that have lost control of their underlying tissue-specific expression patterns or that originated from a minority subpopulation of normal cells whose RPT expression is not representative of the remainder of the tissue.

In addition to their tissue-specific patterning, virtually all tumors showed perturbations of RPT expression that readily allowed them to be distinguished from the normal tissues from which they originated. For some cancers, the tumor-specific patterning of RPT expression was relatively homogeneous and could not otherwise be subcategorized. Most cohorts, however, were comprised of subgroups of tumors with distinct RPT expression patterns, all of which nonetheless remained distinguishable from normal tissue. The fact that many of these patterns correlated with molecular and clinical features implicates RPT expression patterns in tumor biology.

Aside from potentially altering translation, the notion that altered RP expression might influence the behaviors of both normal tissues and tumors is not new. In the ribosomopathies, the binding of any one of about a dozen RPs to MDM2 with subsequent stabilization of TP53 is thought to underlie the bone marrow failure that accompanies these disorders [6, 9, 10]. It has been proposed that subsequent circumvention of this TP53-mediated senescence by mutation and/or dysregulation of the p19^*ARF*^/MDM2/TP53 pathway is responsible for the propensity for eventual neoplastic progression [38]. In cancers, the binding of free RPs to MDM2 has been shown to mediate the response to ribosomal-stress-inducing chemotherapeutics such as actinomycin D and 5-fluorouracil [20, 39, 40].

Individual RPs have also been associated with specific tumor phenotypes. For example, RPL3 expression is a determinant of chemotherapy response in certain lung and colon cancers. RPL3 also associates with the high-risk neuroblastoma subtype and may have a role in the acquisition of lung cancer multidrug resistance [19–21]. Breast cancers with elevated expression of *RPL19* are more sensitive to apoptosis-promoting drugs that induce endoplasmic reticulum stress [13]. *RPS11* and *RPS20* have been proposed as prognostic markers in glioblastoma [16] and the down-regulation of *RPL10* correlates with altered treatment response to dimethylaminoparthenolide (DMAPT) in pancreatic cancer [22].

Our results also significantly extend the findings of previous studies by demonstrating that, in the vast majority of cancers, subsets of RPTs are expressed coordinately and have additional interpretive power when examined in the context of global RPT expression patterning. This suggests that further insights into the roles RPTs have in tumor development may be revealed by evaluating RPT relative expression. For example, the regulation of chemotherapy response by RPL3 described above may be found to occur in other cancer types once the expression of *RPL3* relative to other RPTs has been accounted for. The apparent crucial role of RPT patterning in tumors may explain why a previous study found conflicting results when examining the expression of individual RPs in tumors [14].

Our results suggest a more ubiquitous role for RPL3 in regulating tumor phenotypes, beyond that already described in colorectal carcinoma, lung cancers, and neuroblastoma [19–21]. Of the recurring RPT expression patterns discovered by *t*-SNE, the pattern associated with *RPL3* down-regulation occurred most frequently and involved tumors from nine cancer cohorts. Many clusters of tumors with down-regulated *RPL3*, including HCC, kidney clear cell cancer, and brain cancer, possessed inferior survival. The fact that relative down-regulation of *RPL3* occurred in these tumor clusters with predictable expression of 11 other RPTs suggests that RPL3 may be acting in concert with these other identified RPs to exert its effects.

Other recurrent RPT expression patterns across cancer cohorts involved *RPS4X, RPL13, RPL8* and *RPL30* (Table 1). Altered *RPS4X* expression, found in six cancer cohorts, associated with unique expression of nine other RPTs, strongly suggesting an underlying coordinated expression, the mechanism of which remains to be identified. As with *RPL3*, deregulated *RPS4X* has been previously associated with various tumors and tumor phenotypes, including subgroups of colorectal carcinoma, a myelodysplasia risk signature and poor prognosis in bladder cancer [15, 18, 41]. Interestingly, some of our tumor clusters with altered *RPS4X* expression were comprised of a greater proportion of females than males (Table 1 and Table 3), perhaps reflecting *RPS4X’s* residence on the X chromosome. Although the cause of perturbed *RPS4X* expression in these tumor clusters is unknown, altered methylation patterns on chromosome X have been described in different subsets of cancers [42, 43] and could be responsible for the expression patterns detected by *t*-SNE.

Unlike *RPL3* and *RPS4X*, *RPL13*’s role in tumor development is less clear. RPL13 activation has been described in a subset of gastrointestinal malignancies and correlated with greater proliferative capacity and attenuated chemoresistance [44], but further evidence for a role of RPL13 in tumor development is lacking. Furthermore, clinical correlations of the prostate, uterine and kidney cancer *t*-SNE clusters described here with relative overexpression of *RPL13* were inconsistent. Uterine cancers with high relative *RPL13* expression tended to correlate with favorable survival, whereas prostate cancers with high *RPL13* showed no differences in prognosis or clinical features. In contrast, kidney clear cell carcinomas with high *RPL13* expression tended to be of higher pathologic grade and were associated with significantly poorer survival (Tables 1 and 3, and Fig. 3b). The fact that these clusters shared similar patterning of 42 other RPTs suggests that the inciting factors responsible for higher *RPL13* expression are not only shared by these tumors but coordinately regulate a common subset of RPTs, with different biological outcomes likely reflecting other tissue-specific factors.

In some cases, RPT expression patterns could be accounted for in part by CNVs, as exemplified by the recurrent *RPL8* and *RPL30* overexpression pattern (Tables 1 and 2). Virtually all tumors with this expression pattern possessed co-amplification of a region on 8q22–24 that includes *RPL8*, *RPL30*, and the oncogenes *MYC* and *PVT1.* Amplification of this region has been previously described in breast cancers and correlates with chemoresistance and metastasis [36, 37, 45–47]. Our results indicate that this amplification and the ensuing overexpression of *RPL8* and *RPL30* also occurs in subsets of melanoma, liver, prostate, lung, and head and neck cancers. CNVs of *RPL19* and *RPL23* in breast cancer (Table 2) likely occur due to their co-amplification with *ERBB2* on 17q12. Overexpression of *RPL19* has previously been described in a subset of breast cancers [13]. The small cluster of 144 tumors that did not group according to tissue of origin (Additional file 1: Figure S4), comprised of tumors from 15 cohorts, also shared amplification of this region on 17q12, indicating that this CNV is not restricted to breast cancers and ultimately affects global RPT expression patterning. Amplification of a region on 11q13 that contains *RPS3*, occurring in a cluster of breast cancers and HCCs, has been previously described in both cancers and is thought to confer unfavorable prognosis due to amplification of the adjacent oncogene *EMS1* [48, 49]. The co-deletion of 19q13 along with 1p, which together includes 12 RP genes, has been described in low-grade gliomas and confers a favorable prognosis [50, 51].

The co-overexpression *RPS25* and *RPS4X* detected in one cluster of AML (Fig. 2) has been previously identified as contributing to the poor risk signature in myelodysplastic syndrome [41]. This also associated with significant differential expression of 37 RPTs, which is consistent with our finding that *RPS25* and *RPS4X* overexpression occur within the context of a larger and coordinated pattern of RPT expression. The *RPS25* and *RPS4X* overexpressing AML cases likely possess a similar molecular alteration to those with the poor risk signature in MDS.

Collectively, our findings provide strong evidence to support the notion that RPT regulation by both tumors and normal tissues is complex, ordered, and highly coordinated. Although the means by which altered RPT patterns influence the pathogenesis and/or behavior of tumors remain incompletely understood, several non-mutually exclusive mechanisms can be envisioned. First, changes in RP levels may influence overall ribosome composition, thereby affecting their affinity for certain classes of transcripts and/or the efficiency with which they are translated. One such class of transcripts may be those with IRES elements, *cis*-regulatory sequences found in the 5′-untranslated regions of more than 10% of cellular mRNAs. IRES elements are found with particularly high frequency in transcripts encoding proteins involved in cell cycle control and various types of stress responses. Efficient translation of these IRES-containing transcripts has been shown to depend on specific RPs, notably RPS25*,* RPS19 and RPL11 [52–54]. Changes in ribosome affinity for IRES elements have been shown to reduce translation of tumor suppressors such as p27 and TP53 and to promote cancer development [55].

RPs may also influence cancer development via extra-ribosomal pathways. In addition to their stabilization of TP53 mediated by binding to and inactivating MDM2, specific RPs have been shown to inactivate Myc; to inhibit the Myc target Lin28B; to activate NF-κB, cyclins, and cyclin-dependent kinases and to regulate a variety of other tumorigenic functions and immunogenic pathways [4, 5].

In addition to providing evidence that tumors may use RPs to direct tumor phenotypes, our findings have allowed us to leverage the tissue- and tumor-specificity of RPT expression to generate highly sensitive and specific models that allow for precise tumor identification and sub-classification (Additional file 1: Table S2). Clinically, these might be useful for determining the tissue of origin of undifferentiated tumors and for predicting long-term behaviors in otherwise homogeneous cancers such as kidney clear cell carcinoma and those of the central nervous system (Fig. 3b). With more samples and further refinement to ANN structures, future iterations of these models will likely have even greater discriminatory power.

A limitation of using data from TCGA is the fact that transcript expression does not always correlate with protein expression, particularly in cancers [56–58]. Thus, it is difficult to predict how the different tissue-specific RPT expression patterns we identified correlate with actual protein expression in these cancers and/or with the numerous post-translational modifications that can alter RP behaviors [59, 60]. As this is a cross-sectional study, we also recognize that causality cannot be inferred, and it remains unknown whether altered RPT expression is an early or late event in tumorigenesis despite its predictive value. Furthermore, while RPT expression patterns appear to have significant predictive value in the large dataset we have analyzed, further cross-validation with additional transcriptional data in both primary tumors and metastatic lesions will be important in confirming potential clinical utility. Finally, additional molecular analyses of the identified *t*-SNE clusters with whole-transcriptome sequencing data, pathway analysis, whole-genome DNA mutation data, and DNA methylation patterning may offer additional insights into the biological mechanisms that link altered RPT expression with tumor phenotypes.

## Conclusions

In summary, machine learning-based approaches have allowed us to show unequivocally that RPTs are expressed in distinct patterns across tissue types. This tissue-specificity persists in tumors, yet normal tissues and tumors can be readily distinguished from one another with high degrees of accuracy and confidence. Many cancers can be further sub-categorized into heretofore unrecognized, yet clinically important, subtypes based only upon RPT expression patterns. Several patterns of RPT expression recur across cancer types, suggesting common underlying modes of transcriptional regulation. Our results indicate that the expression of RPTs in tumors is biologically coordinated, clinically meaningful, and can be leveraged to create potential clinical tools for tumor classification and therapeutic stratification.

## Additional file

Additional file 1: Supplementary Information. Contains Figure S1-S5, Table S1-S2, and their respective legends. (DOCX 1621 kb)

## Abbreviations

ANOVA: Analysis of variance
CNV: Copy number variation
DBA: Diamond-blackfan anemia
IRES: Internal ribosome entry site
LR: Logistic regression
MDS: Myelodysplastic syndrome
RP: Ribosomal protein
RPKM: Reads-per-kilobase-million
RPT: Ribosomal protein transcript
TCGA: The cancer genome atlas
t-SNE: t-distributed stochastic neighbor embedding
uORF: Upstream open reading frame
