# Supplementary material for: Diagnostic and prognostic implications of ribosomal protein transcript expression patterns in human cancers
Source: BMC Cancer. 2018 Mar 12;18:275. doi: 10.1186/s12885-018-4178-z (PMC5848553; doi:10.1186/s12885-018-4178-z)
Supplement: Supplementary file 1 — Supplementary Information. Contains Figure S1-S5, Table S1-S2, and their respective legends. (DOCX 1621 kb) [file 12885_2018_4178_MOESM1_ESM.docx]

**Supplementary Information for:**

**Diagnostic and Prognostic Implications of Ribosomal Protein**

**Transcript Expression Patterns in Human Cancers**

James M. Dolezal, Arie P. Dash, Edward V. Prochownik

This Supplementary Information includes:

Supplementary Figures 1 – 5

Supplementary Figure Legends

Supplementary Tables 1-2

Supplementary Table Legends

**
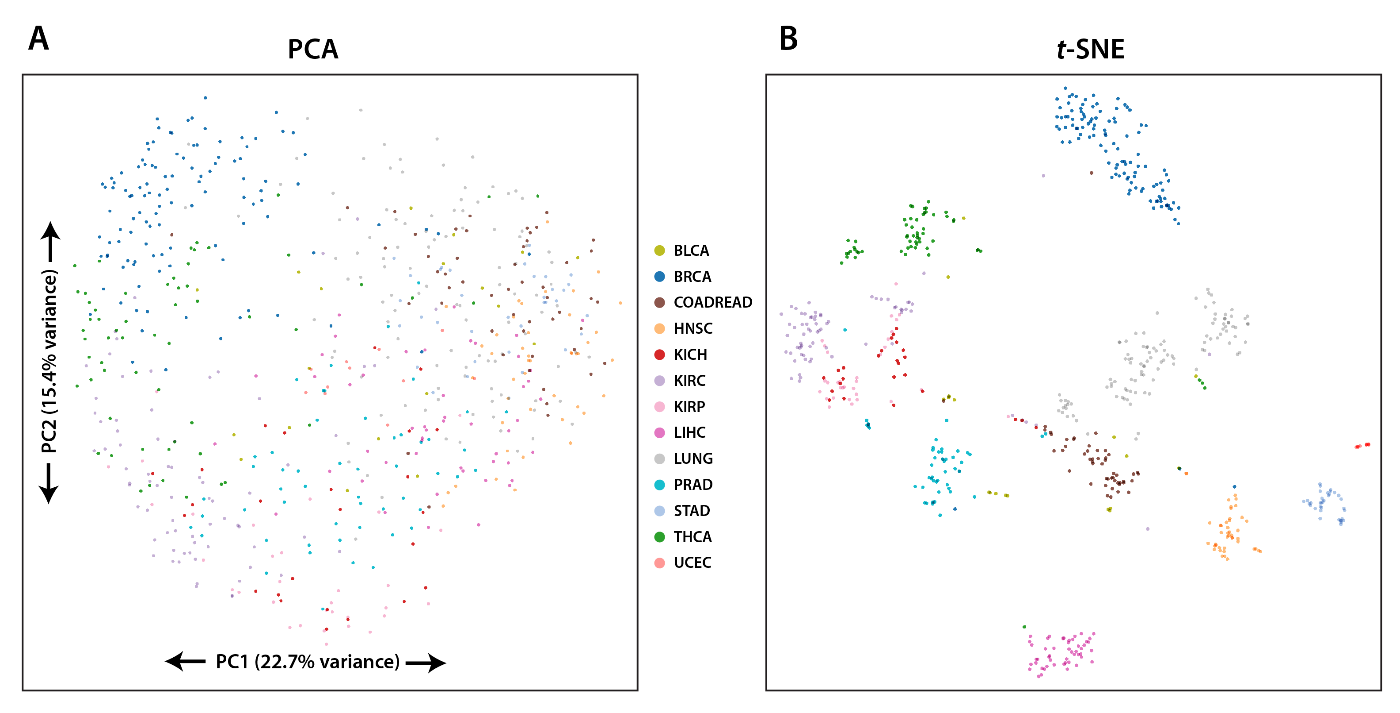
**

**Figure S1. Normal tissues cluster distinctly with *t*-SNE.** RPT expression in normal tissue samples from cohorts with at least 10 normal tissues was visualized with two dimensionality reduction techniques, PCA (A) and *t*-SNE (B). Using PCA, normal tissue samples exhibit slight clustering according to tissue type, but differences in RPT expression between cohorts are not distinct. With *t*-SNE, normal tissues cluster according to tissue type nearly perfectly. Note that overlap occurs between samples from kidney chromophobe (KICH), kidney clear cell carcinoma (KIRC) and kidney papillary cell carcinoma (KIRP) due to the fact that normal tissues are all kidney in these cohorts. The esophageal cancer cohort  (Yong et al.) was excluded from this graph, as data were missing expression of five RPTs – *RPL17*, *RPL36A*, *RPS10*, *RPS17*, and *RPS4Y1*. Parameters used for *t*-SNE: perplexity = 31, learning rate = 10, iterations = 5000.


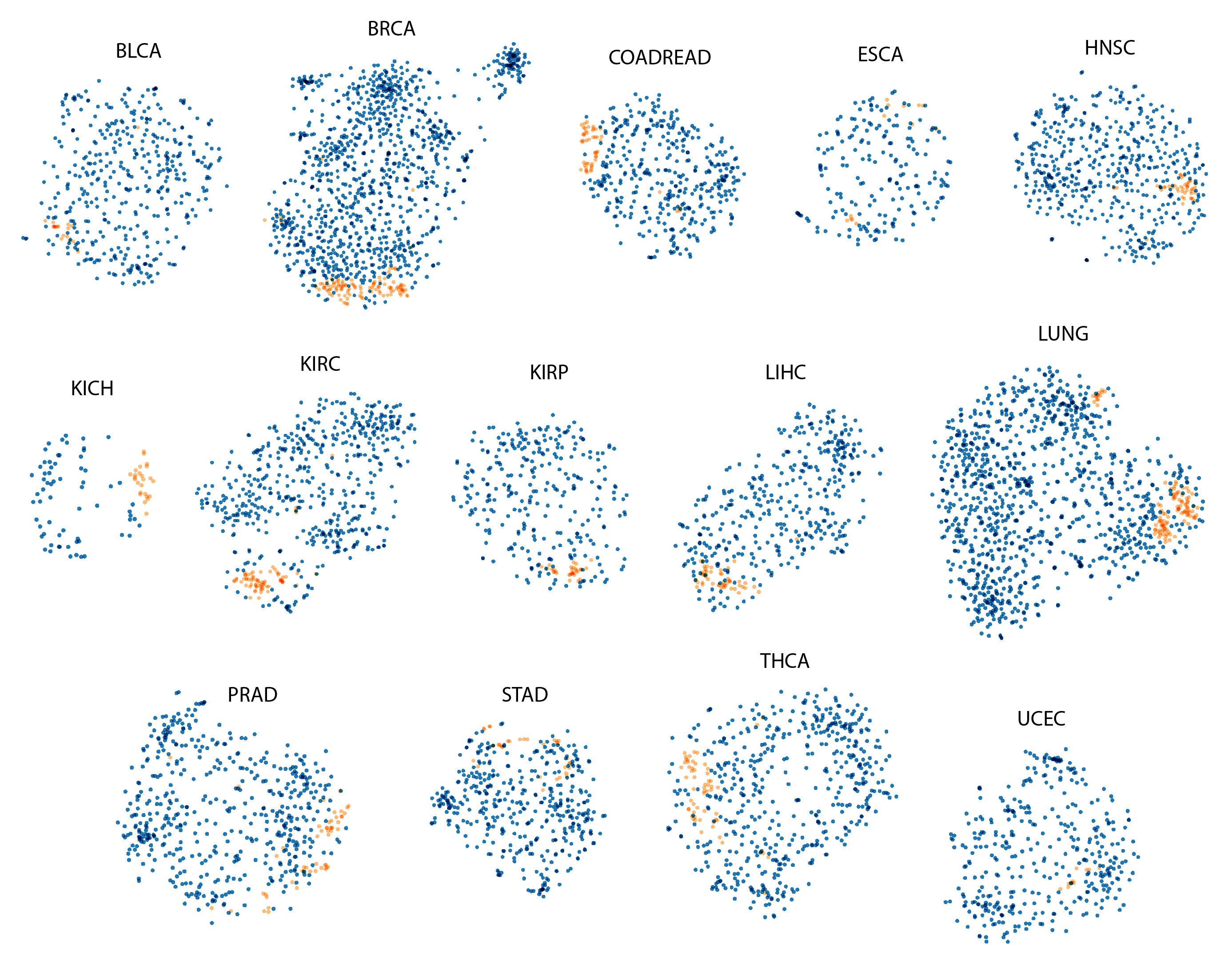
**Figure S2. Normal tissues cluster distinctly from tumors of the same tissue type.** RPT expression of both normal tissue and tumor samples were analyzed with *t*-SNE in all cohorts with at least 10 normal tissue samples. Tumors are colored blue, and normal tissues are colored orange. Normal tissues sub-cluster together distinctly from tumors but within the larger tumor cluster. Thus, RPT expression in tumors is similar to, but distinct from, normal tissues, and tumors have greater overall heterogeneity in their RPT expression patterns. *t*-SNE parameters for all plots: perplexity = 60, learning rate = 10, iterations = 2000.

**
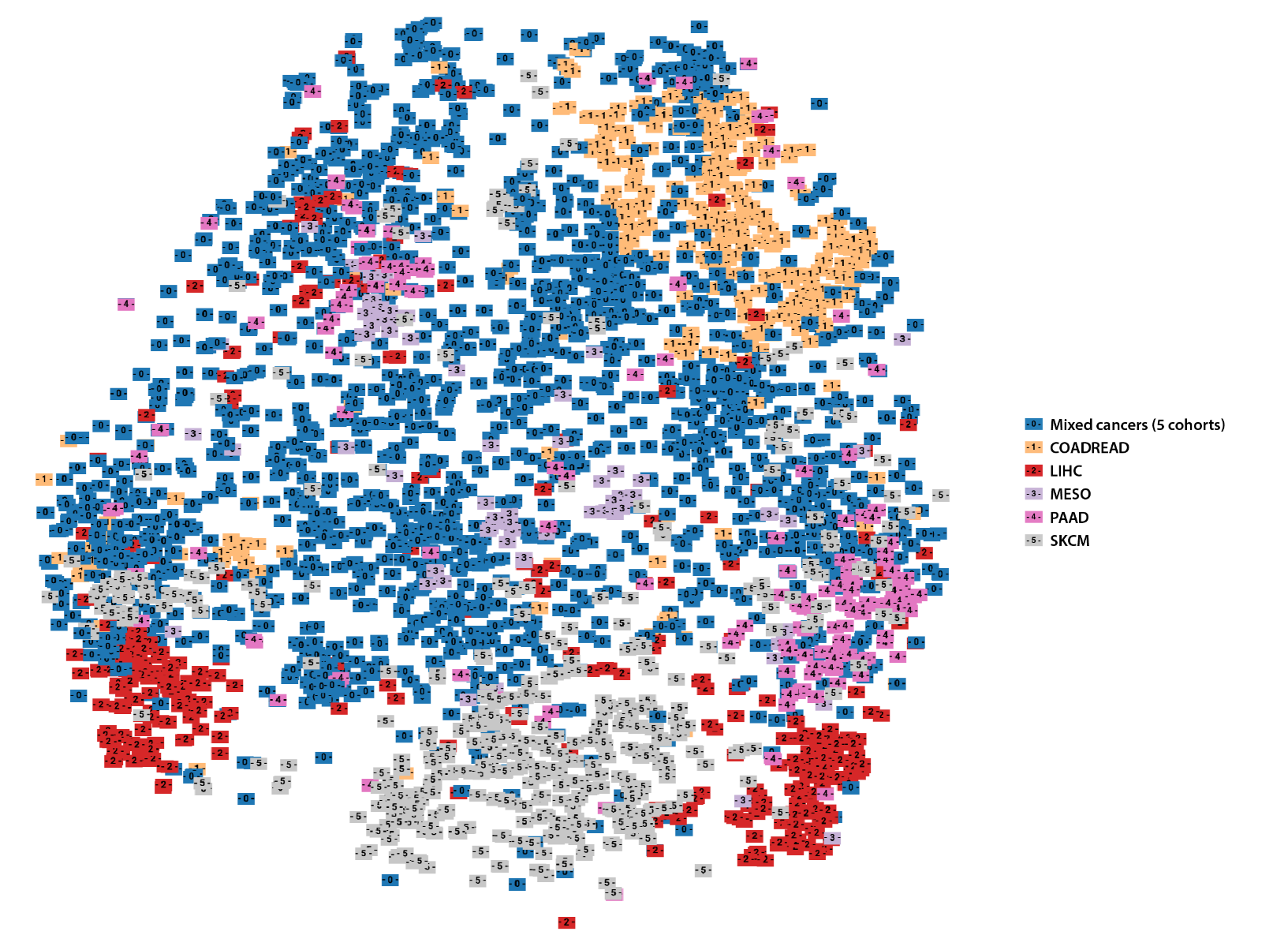
**

**Figure S3. Tumor cohorts with overlapping RPT expression profiles.** Five cancer cohorts were comprised of tumors with overlapping RPT expression patterns and did not cluster distinctly with *t*-SNE. These cohorts – cholangiocarcinoma (CHOL), lung (LUNG), bladder (BLCA), cervical (CESC), and uterine carcinosarcoma (UCS) – were grouped together, here referred to as “mixed cancers.” This group of mixed cancers displayed significant overlap with five other cohorts that otherwise clustered with fair distinction from one another – colorectal (COADREAD), liver (LIHC), mesothelioma (MESO), pancreatic (PAAD), and skin cutaneous melanoma (SKCM). These five cohorts were analyzed alongside the mixed cancer group with *t*-SNE with the results shown here. The following *t*-SNE parameters were used: perplexity = 24, learning rate (epsilon) = 10, iterations = 5000.

**
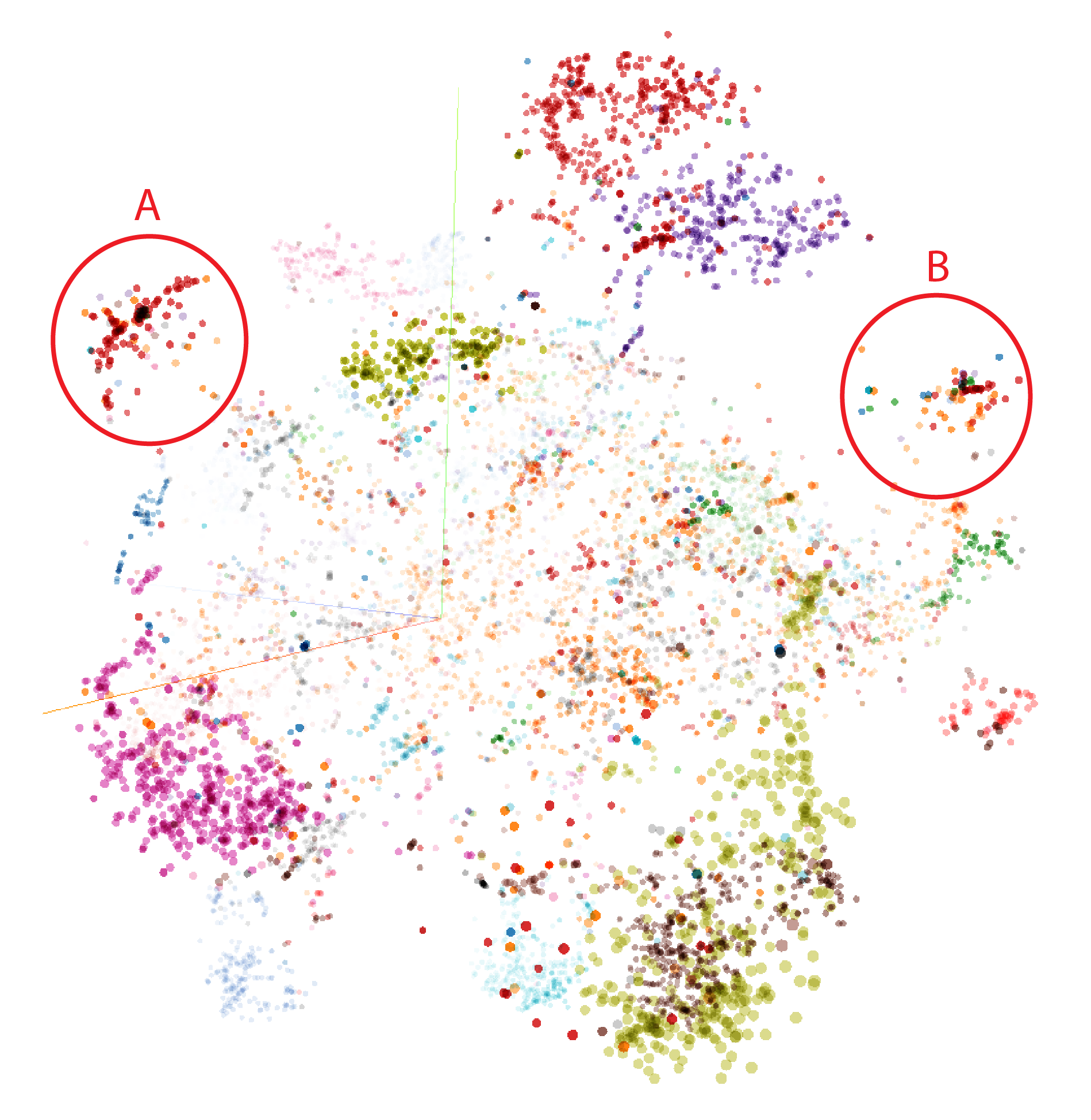
Figure S4. Pan-cancer *t*-SNE plot reveals tumor clusters not associating with tissue of origin.** Three-dimensional *t*-SNE analysis of RPT expression in tumors from 29 cancer cohorts. Tumors from ESCA were excluded from this pan-cancer analysis due to the missing expression of five RPTs: *RPL17*, *RPL36A*, *RPS10*, *RPS17*, and *RPS4Y1*. In addition to the numerous clusters associated with tumor type, two clusters were identified that did not associate with tissue of origin. Both are circled here in red. The first, labeled “A,” was comprised of 143 tumors, all of which shared relative up-regulation of *RPL19* and *RPL23*, along with amplification of a region on 17q12 containing the genes *RPL19*, *RPL23*, and *ERBB2* (Her2/Neu). These tumors were from the following cohorts: BLCA, BRCA, CESC, COADREAD, HNSC, LUNG, PAAD, SKCM, STAD, KIRC, KIRP, OV, THYM, UCEC, and UCS. The second cluster, labeled “B,” was comprised of 77 tumors, and no discernable shared RPT expression pattern could be identified in this group. These tumors were from the cohorts BLCA, BRCA, CESC, COADREAD, HNSC, LUNG, OV, PAAD, SARC, SKCM, TGCT, and UCS.

**
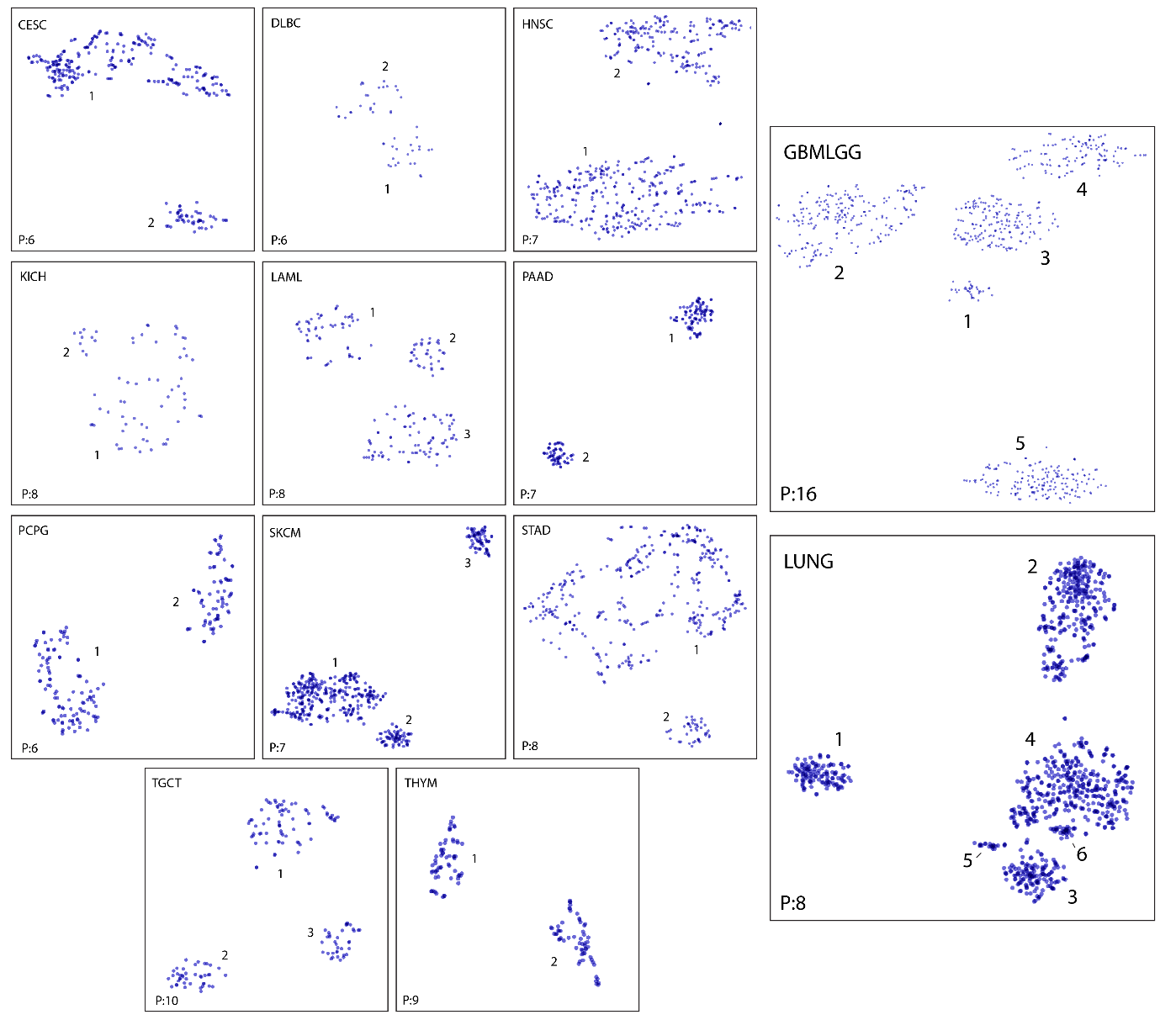
**

**Figure S5. Sub-clustering of RPT expression patterns in additional tumor cohorts.** *t*-SNE plots of tumor RPT expression patterns in 13 cohorts with sub-clusters, in addition to those already displayed in **Figure 1D**. Perplexity settings for *t*-SNE analyses are designated in each plot by “P:”. All analyses were performed with learning rate (epsilon) = 10 and iterations = 5000.

**Table S1.** The Cancer Genome Atlas (TCGA) cohorts and clusters identified by *t*-SNE.

| **Abbr** | **Cancer type** | **Tumors** | **Normal tissues** | **Tumor clusters** |
| --- | --- | --- | --- | --- |
| **BLCA** | Bladder cancer | 407 | 19 | - |
| **BRCA** | Breast cancer | 1097 | 114 | 4 |
| **COADREAD** | Colorectal carcinoma | 380 | 51 | - |
| **LUNG** | Combined lung cancer | 1017 | 110 | 6 |
| **UCEC** | Uterine corpus endometrial carcinoma | 370 | 11 | 3 |
| **GBMLGG** | Glioblastoma multiforme and low-grade glioma | 670 | 5 | 5 |
| **HNSC** | Head and neck cancer | 520 | 44 | 2 |
| **LIHC** | Hepatocellular carcinoma | 371 | 50 | 3 |
| **KIRC** | Kidney clear cell carcinoma | 533 | 72 | 3 |
| **KIRP** | Kidney papillary cell carcinoma | 290 | 32 | - |
| **PRAD** | Prostate cancer | 497 | 52 | 3 |
| **STAD** | Stomach cancer | 415 | 35 | 2 |
| **THCA** | Thyroid carcinoma | 505 | 59 | 3 |
| **LAML** | Acute myeloid leukemia | 173 | 0 | 3 |
| **ESCA** | Esophageal carcinoma | 184 | 13 | - |
| **CHOL** | Bile duct cancer | 36 | 9 | - |
| **CESC** | Cervical cancer | 303 | 3 | 2 |
| **SKCM** | Melanoma | 472 | 1 | 3 |
| **MESO** | Mesothelioma | 87 | 0 | - |
| **UVM** | Ocular melanomas | 80 | 0 | - |
| **OV** | Ovarian cancer | 303 | 0 | - |
| **PAAD** | Pancreatic cancer | 178 | 4 | 2 |
| **PCPG** | Pheochromocytoma and paraganglioma | 178 | 3 | 2 |
| **SARC** | Sarcoma | 259 | 2 | - |
| **TGCT** | Testicular cancer | 150 | 0 | 3 |
| **THYM** | Thymoma | 120 | 2 | 2 |
| **UCS** | Uterine carcinosarcoma | 57 | 0 | - |
| **ACC** | Adrenocortical cancer | 78 | 0 | - |
| **KICH** | Kidney chromophobe | 66 | 25 | 2 |
| **DLBC** | Large B-cell lymphoma | 48 | 0 | 2 |

**Table S1. The Cancer Genome Atlas (TCGA) cohorts and clusters identified by t-SNE.** Relative expression of RPTs was calculated using RNA-seq expression data from TCGA, accessed via the UC Santa Cruz Xenabrowser. Clustering of RPT expression was investigated with *t*-SNE using Tensorflow, with perplexity varying between 6-15. Exact parameters used for final *t*-SNE plots can be found in the respective figures (**Figure 1D** and **Supplementary Figure 5**). Clusters were defined as groups of >10 tumors visually separating into distinct clusters (**Figure 2A**). Nineteen cancer cohorts demonstrated distinct clustering by *t*-SNE. Cancer cohorts without sub-clustering are denoted with “-“.

**Table S2.** Logistic regression (LR) and Artificial Neural Network (ANN) models classify tumors by RPT expression.

| **Classification** | **Type** | **Criteria** | **Number**  **of Cohorts** | **Number**  **of Samples** | **Test set size** | **Accuracy** | ***c*-statistic (LR)** | **Sensitivity**  **(LR)** | **Specificity (LR)** |
| --- | --- | --- | --- | --- | --- | --- | --- | --- | --- |
| **Diagnostic**:  Cancer type | ANN | >75 tumors/cohort | 25 | 1950 | 585 | 93.2% | - | - | - |
| **Diagnostic**:  Tumor v. normal | LR | >25 normals/cohort | 11 | 550 | - | 98.4% | 0.999 | 98.2% | 98.6% |
| **Diagnostic**: distinguish GBM from LGG | LR | GBMLGG tumors | 1 | 670 | - | 100% | 1.0 | 100% | 100% |
| **Prognostic**: classify by  LGG cluster | ANN | LGG tumors | 1 | 516 | 155 | 94.8% | - | - | - |
| **Prognostic**: identify  KIRC Cluster 2 | LR | KIRC tumors | 1 | 533 | - | 95.3% | 0.986 | 94.8% | 95.6% |
| **Prognostic**: identify  KIRC Cluster 3 | LR | KIRC tumors | 1 | 533 | - | 97.4% | 0.996 | 96.4% | 98.1% |
| **Prognostic**: identify  UCEC Cluster 1 | LR | UCEC tumors | 1 | 533 | - | 96.8% | 0.996 | 95.0% | 97.8% |

**Table S2. Logistic regression (LR) and Artificial Neural Network (ANN) models classify tumors by RPT expression.** Using RPT expression, various models were constructed to predict features identified by the previous *t*-SNE analyses. ANNs were constructed with Tensorflow and trained on 60% of data, with 10% of data saved for validation during hyper-parameter tuning. For ANNs, “accuracy” reflects classification accuracy of the final chosen model after hyper-parameter tuning on a separate test set, comprised of 30% of the original data. All data for ANN training and testing was balanced by cancer cohort to reduce the risk of bias, such that the same number of samples from each cohort were included in training and testing. LR models were constructed using Stata SE.
